# Expression of concern: Potential of non-thermal discharge plasmas for activated sludge settling: effects and underlying mechanisms

**DOI:** 10.1039/d6ra90037b

**Published:** 2026-04-07

**Authors:** Yun Chen, Siqi Liu, Zhiyin Ren, Qi Wang, Ying Zhang, Yajie Zuo, Jian Zhou, Hongtao Jia, Tiecheng Wang

**Affiliations:** a Ningxia Houde Environmental Protection Technology Co., Ltd Yinchuan 750000 China; b College of Information Science and Technology, Nanjing Forestry University Nanjing 210037 China; c College of Natural Resources and Environment, Northwest A&F University Yangling Shaanxi Province 712100 PR China wangtiecheng2008@126.com; d Key Laboratory of Plant Nutrition and the Agri-environment in Northwest China, Ministry of Agriculture Yangling Shaanxi 712100 PR China; e College of Resources and Environment, Xinjiang Agricultural University Urumqi 830052 China

## Abstract

Expression of concern for ‘Potential of non-thermal discharge plasmas for activated sludge settling: effects and underlying mechanisms’ by Yun Chen *et al.*, *RSC Adv.*, 2023, **13**, 19869–19880, https://doi.org/10.1039/D3RA02921B.

The Royal Society of Chemistry is publishing this expression of concern in order to alert readers that concerns have been raised regarding the integrity of the SEM images in Fig. 6, the EPR data in Fig. 9b and the data in Fig. 10.

The Royal Society of Chemistry has asked the affiliated institution (Northwest A&F University, China) to investigate this matter and confirm the integrity and reliability of the published data.

An expression of concern will continue to be associated with the article until we receive conclusive evidence regarding the reliability of the reported data.

Laura Fisher

24th March 2026

Executive Editor, *RSC Advances*

